# Genomic signatures define three subtypes of *EGFR*-mutant stage II–III non-small-cell lung cancer with distinct adjuvant therapy outcomes

**DOI:** 10.1038/s41467-021-26806-7

**Published:** 2021-11-08

**Authors:** Si-Yang Liu, Hua Bao, Qun Wang, Wei-Min Mao, Yedan Chen, Xiaoling Tong, Song-Tao Xu, Lin Wu, Yu-Cheng Wei, Yong-Yu Liu, Chun Chen, Ying Cheng, Rong Yin, Fan Yang, Sheng-Xiang Ren, Xiao-Fei Li, Jian Li, Cheng Huang, Zhi-Dong Liu, Shun Xu, Ke-Neng Chen, Shi-Dong Xu, Lun-Xu Liu, Ping Yu, Bu-Hai Wang, Hai-Tao Ma, Hong-Hong Yan, Song Dong, Xu-Chao Zhang, Jian Su, Jin-Ji Yang, Xue-Ning Yang, Qing Zhou, Xue Wu, Yang Shao, Wen-Zhao Zhong, Yi-Long Wu

**Affiliations:** 1 Guangdong Lung Cancer Institute, Guangdong Provincial People’s Hospital, and Guangdong Academy of Medical Sciences, School of Medicine, South China University of Technology, Guangzhou, China; 2 Nanjing Geneseeq Technology Inc., Nanjing, China; 3 Fudan University Affiliated Zhongshan Hospital, Shanghai, China; 4 Zhejiang Cancer Hospital, Hangzhou, China; 5 Hunan Cancer Hospital, Changsha, China; 6 The Affiliated Hospital of Medical College Qingdao University, Qingdao, China; 7 Shenyang Chest Hospital, Shenyang, China; 8 Fujian Medical University Union Hospital, Fuzhou, China; 9 Jilin Provincial Tumor Hospital, Changchun, China; 10 Jiangsu Cancer Hospital, Nanjing, China; 11 The People’s Hospital of Peking University, Beijing, China; 12 Shanghai Pulmonary Hospital, Shanghai, China; 13 Tangdu Hospital, Xi’an, China; 14 Peking University First Hospital, Beijing, China; 15 Fujian Cancer Hospital, Fuzhou, China; 16 Beijing Chest Hospital, Beijing, China; 17 The First Hospital of China Medical University, Shenyang, China; 18 Beijing Cancer Hospital, Beijing, China; 19 Harbin Medical University Cancer Hospital, Harbin, China; 20 West China Hospital of Sichuan University, Chengdu, China; 21 Sichuan Cancer Hospital, Chengdu, China; 22 The Northern Jiangsu People’s Hospital, Yangzhou, China; 23 The First Affiliated Hospital of Suzhou University, Suzhou, China; 24 School of Public Health, Nanjing Medical University, Nanjing, China

**Keywords:** Cancer genomics, Targeted therapies, Non-small-cell lung cancer, Predictive markers, Surgical oncology

## Abstract

The ADJUVANT study reported the comparative superiority of adjuvant gefitinib over chemotherapy in disease-free survival of resected *EGFR*-mutant stage II–IIIA non-small cell lung cancer (NSCLC). However, not all patients experienced favorable clinical outcomes with tyrosine kinase inhibitors (TKI), raising the necessity for further biomarker assessment. In this work, by comprehensive genomic profiling of 171 tumor tissues from the ADJUVANT trial, five predictive biomarkers are identified (*TP53* exon4/5 mutations, *RB1* alterations, and copy number gains of *NKX2-1*, *CDK4*, and *MYC*). Then we integrate them into the Multiple-gene INdex to Evaluate the Relative benefit of Various Adjuvant therapies (MINERVA) score, which categorizes patients into three subgroups with relative disease-free survival and overall survival benefits from either adjuvant gefitinib or chemotherapy (Highly TKI-Preferable, TKI-Preferable, and Chemotherapy-Preferable groups). This study demonstrates that predictive genomic signatures could potentially stratify resected *EGFR*-mutant NSCLC patients and provide precise guidance towards future personalized adjuvant therapy.

## Abstract (web summary)

Adjuvant gefitinib improves outcomes in non-small cell lung cancer (NSCLC) patients compared to chemotherapy, but not in all cases. Here, the authors find genomic biomarkers of response to gefitinib in NSCLC patients from the ADJUVANT trial, and propose a score to stratify them by potential benefit from the treatment.

## Introduction

Cisplatin-based adjuvant chemotherapy currently constitutes the standard-of-care after curative surgery for stage IIA-IIIB resected non-small cell lung cancer (NSCLC)^1,2^. However, the 5-year survival rate still remains unsatisfactory, with alarming levels of grade 3 toxicity observed in more than 60% of the patients^3^. Hence, alternative adjuvant regimens with epidermal growth factor receptor (EGFR) tyrosine kinase inhibitors (TKI) have been studied through several prospective trials^4,5^. The randomized phase III ADJUVANT study has actually presented significant prolonged disease-free survival (DFS) in *EGFR*-mutant NSCLC, after adjuvant gefitinib, as compared to the DFS after chemotherapy with vinorelbine and cisplatin (VP)^6^. Two phase 2 trials, SELECT and EVAN, have shown improved 2-year DFS with erlotinib. Early revelations of the ADAURA trial also presented remarkable improvements of DFS with the third generation EGFR-TKI, osimertinib^7–9^. However, approximately 19% to 40% of TKI-treated patients still relapse after these trials^6,8^, suggesting the inadequacy of *EGFR*-sensitizing mutants alone as a biomarker for adjuvant treatment selection.

The mixed clinical responses of NSCLC with targeted therapy can be attributed to molecular heterogeneity caused by different clonal populations, aggregated in a particular tumor, undergoing stage-specific evolution. Resulting selective pressure then further induces subclonal mutations, and promotes tumor expansion^10–13^. The most prevalent co-mutations, such as alterations in the *TP53*, *RB1* and *NKX2-1*, usually cooperate to promote a local growth advantage, and support clonal expansion throughout tumor development^10,11,14^. In advanced *EGFR* mutant NSCLC, tumors with concurrent *TP53* or *RB1* mutations then further disrupted genome stability and exerted higher risks for histological transformation and TKI resistance^15^. In addition to gene level alterations, co-mutations on the exon levels can also affect patient outcomes^16^.

As early-stage NSCLC also shows a high degree of intratumor heterogeneity with divergent evolutionary lineages^17^, the established norm of estimating only a single driver oncogene through randomized trials for adjuvant targeted therapies fails to address the underlying complications of intratumor molecular heterogeneity. In this regard, the development of next-generation sequencing (NGS) technology has accelerated the analysis and integration of huge bulks of genomic signatures, thereby increased the focus on developing multi-gene predictive models for therapeutic decisions^18,19^. Currently, in most single-armed cohort studies, biomarkers were analyzed for their prognostic effects by comparing survival differences between mutant and wildtype patients. However, the more challenging question is whether these biomarkers result in distinct outcomes under different treatments to ultimately guide therapeutic decisions. Therefore, it is important to distinguish predictive markers from the prognostic ones at first. The frequently used term “predict the prognosis” in many biomarker studies may confuse readers of the accurate definition for these two types of biomarkers. Specifically, a predictive biomarker differentiates treatment-specific survival benefits in biomarker-positive or biomarker-negative patients^20^ and further improves patients’ treatment outcomes, while a prognostic biomarker discriminates good or poor survival of patients regardless of treatment. For example, aberrations in the tumor suppressor *TP53* gene are known to correlate with worse prognosis comparing to *TP53* wildtype cancers^16,21^.

Moreover, to reduce the complications in choosing the appropriate statistical tests, a standard and reliable analytical method has been endorsed by Rothwell^22^ and applied in numerous studies^23,24^. As suggested, testing subgroup-treatment effect interaction is a prerequisite in reporting the predictive significance other than subjective observations of the survival curves. Subsequently, a linear discriminant using summation of all predictive values over the set of selected biomarkers is usually adopted for composite score development^25^.

In this study, we conduct a thorough explorative analysis of cancer-related genes through NGS of tumor tissues from the *EGFR*-mutant patients of the ADJUVANT trial, in an attempt to address important co-mutations and identify key predictive biomarkers for adjuvant treatment. We also integrate them into a robust predictive score that can categorize patients into subgroups with distinct survival benefits under either adjuvant gefitinib, or chemotherapy for precision care.

## Results

### Identification of predictive biomarkers from differential DFS

**Fig. 1:**
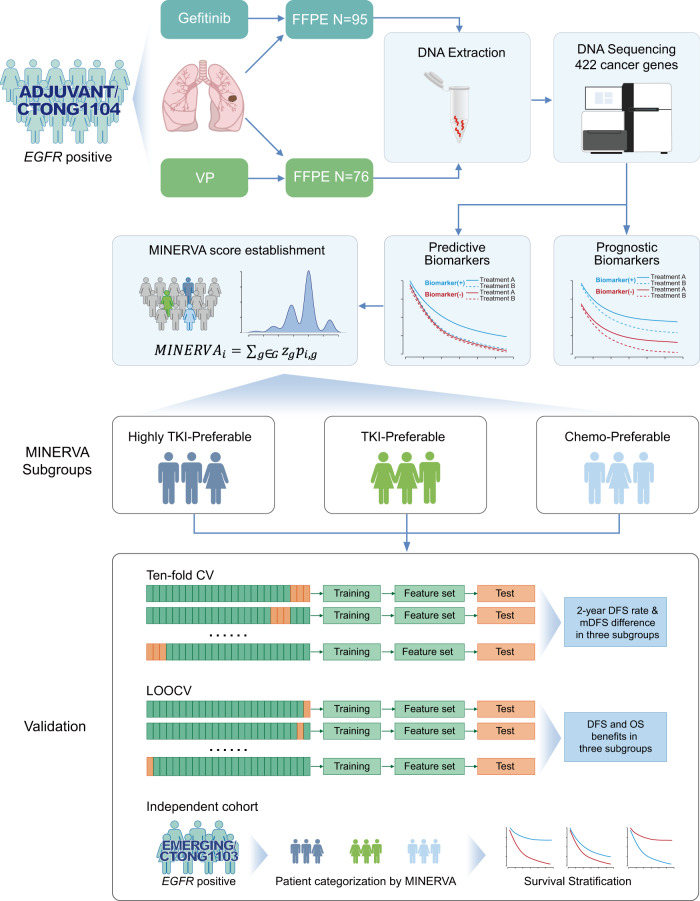
Schematic diagram of patient screening, sample collection, and methodology for developing the clinical predictive model. Formalin-fixed paraffin-embedded (FFPE) samples of patients treated with adjuvant gefitinib or intravenous vinorelbine plus cisplatin (VP) in the ADJUVANT trial were collected for NGS-sequencing. Genomic alterations were analyzed for being predictive or prognostic biomarkers for adjuvant treatment. Predictive markers were selected to develop the Multiple-gene INdex to Evaluate the Relative benefit of Various Adjuvant therapies (MINERVA) score and validated through ten-fold cross validation (CV) or leave-one-out CV (LOOCV) procedures and an independent cohort.

Total 171 patients from the ADJUVANT trial with available baseline surgical specimens have been enrolled for genomic profiling (Fig. 1). The basic characteristics of the patients included in this exploratory cohort have been summarized in Supplementary Table 1. Comprehensive genomic profiling of 422 cancer-related genes revealed comparable frequencies of the highest mutated genes between the two treatment groups (Supplementary Fig. 1). *EGFR* 19del (49% vs. 45%), L858R (47% vs. 53%), and copy number gain (CN gain, 17% vs. 26%) were equally distributed in the adjuvant gefitinib and VP groups. Other co-mutations, including *TP53* (70% vs 64%), *MCL1* (30% vs 16%), *RB1* (25% vs 15%), *NKX2-1* (20% in both), *CDKN2A* (16% vs 19%), *PIK3CA* (14% vs 17%), *MDM2* (14% vs 9%), and *CTNNB1* (7% vs 18%) also presented similar frequencies between the two cohorts. Of note, total 76/171 (44%) patients carried *TP53* DNA binding domain missense mutations (exons 4–8). However, co-drivers frequently found in advanced diseases, e.g. *BRAF* mutations, amplifications of *ERBB2*, or *MET*^13,14,26^, were not as prevalent in our early-stage cohort.

We adopted the popular approach of testing DFS-based gene-by-treatment interaction effects to identify predictive genetic biomarkers for guiding treatment selection^22,27^. Under this test, predictive biomarkers would show different treatment effect for biomarker-positive patients compared to the biomarker-negative population (Supplementary Fig. 2)^20^. We evaluated the predictive power of each mutated gene, and identified the following five predictive markers with significant treatment interactions (Table 1 and Methods): *RB1* alterations [interaction hazard ratio (iHR) 4.07, 95% confidence interval (CI) 1.56–10.58, *P* = 0.004], *NKX2-1* CN gain [iHR 0.26 (95% CI 0.10–0.68), *P* = 0.006], *CDK4* CN gain [iHR 0.14 (95% CI 0.03–0.77), *P* = 0.024], *TP53* exon4/5 missense mutations [iHR 0.33 (95% CI 0.12–0.93), *P* = 0.035], and *MYC* CN gain (iHR 0.10 (95% CI 0.01–0.98), *P* = 0.048). Here, negative iHR indicated relative better survival with adjuvant TKI while positive iHR indicated relative benefit with adjuvant chemotherapy. Importantly, the treatment interactions remained significant for these five predictors even after adjusting for clinical parameters (Supplementary Table 2). The negative adjuvant TKI predictor, *RB1* alterations, combined *RB1* mutations and *RB1* CN loss, since they were functionally similar and both presented marginal significance of treatment interaction due to small sample size of each category (Supplementary Table 3). Besides, as missense mutations on different *TP53* exons might show distinct prognostic or predictive effects^16,28^, these exons were analyzed separately. Like *RB1* alterations, both *TP53* exon 4 and 5 missense mutations (but not exons 6–8) showed marginal significance for treatment interactions and were therefore combined as a single predictive factor. Further, for prognostic analysis, we found that *TP53* exon4/5 missense mutations [multivariate HR 2.69 (95% CI 1.60–4.52), *P* < 0.001] and *TP53* nonsense mutations [multivariate HR 1.69 (95% CI 1.08–2.65, *P* = 0.022)] were both significantly correlated with worse outcomes irrespective of treatment arms, in concordance with *TP53* as a factor for negative prognosis (Supplementary Figs. 2 and 3a, b). Other genetic aberrations that were significantly associated with prognosis were summarized in Supplementary Figure 3 and Supplementary Table 4.

**Table 1:** Predictive values of different genomic aberrations derived according to disease-free survival (DFS).

Predictive markers (treatment-by-gene interaction)				
Mutation subgroup	Recurrence events/no. of patients	iHR^a^ (95% CI^b^)	z-score	*P* Value^d^
*RB1* alterations	23/33	4.07 (1.56–10.58)	2.88	0.004
*NKX2-1* CN^c^ gain	23/34	0.26 (0.10–0.68)	−2.72	0.006
*CDK4* CN^c^ gain	7/12	0.14 (0.03–0.77)	−2.26	0.024
*TP53* exon4/5 missense mutations	20/29	0.33 (0.12–0.93)	−2.11	0.035
*MYC* CN^c^ gain	7/15	0.10 (0.01–0.98)	−1.98	0.048

^a^ iHR, interaction hazard ratio between treatments and gene alterations.

^b^CI, confidence interval.

^c^CN, copy number.

^d^Two-sided P-values of the wald test.

### Integrated MINERVA score via genomic signature

**Fig. 2:**
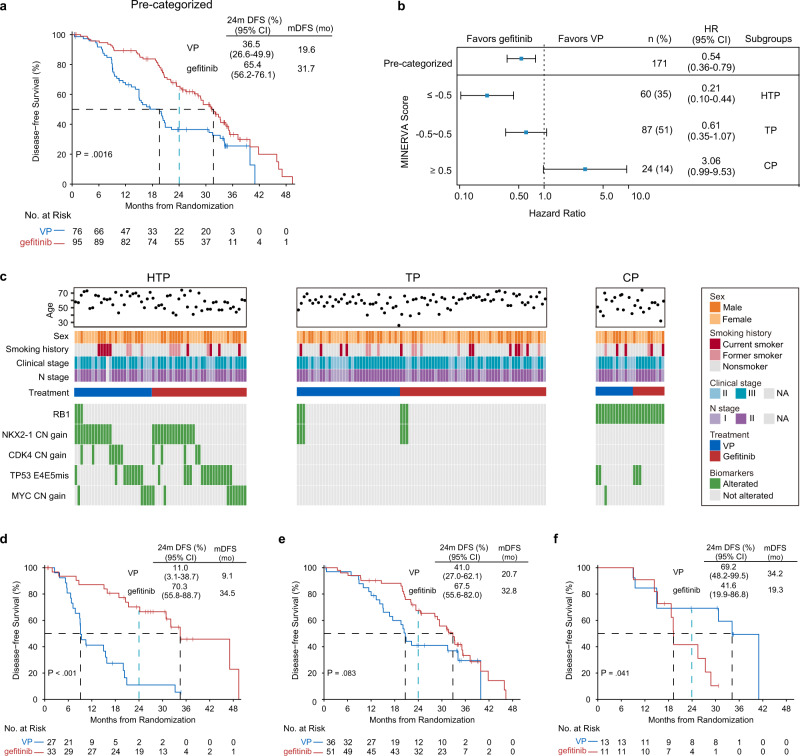
Disease-free Survival (DFS) as per MINERVA subgroups. **a** Kaplan–Meier curves estimate DFS of the pre-categorized cohort which received adjuvant gefitinib or VP treatment (*N* = 171). Two-sided *P* value was calculated using the log-rank test. **b** Forest plot showing the treatment-by-interaction hazard ratio (iHR) of DFS with the cox regression model in subgroups (HTP, highly TKI-preferable group; TP, TKI-preferable group; CP, chemo-preferable group) as classified by MINERVA score. Error bars indicate 95% confidence intervals of the iHRs. **c** Clinical characteristics and genetic alteration spectrums of five predictive biomarkers in three MINERVA subgroups. **d**–**f** Kaplan–Meier curves of DFS for patients treated by adjuvant gefitinib or VP in three MINERVA subgroups. Black dotted lines indicate median DFS. Blue doted lines indicate 2-year survival rates (24 months). Two-sided *P* values were derived from the log-rank test. Exact statistical significance of DFS difference in the HTP group was 2.47 × 10^−5^.

Each of the five biomarkers individually can predict the treatment outcomes for patient subgroups harboring each specific genetic alteration, although, a multigene signature integrating all mutational events at patient level is essential for estimating a patient’s overall response to the molecular heterogeneity of early-stage NSCLC. We, therefore, constructed a MINERVA score to quantitatively assess individual tumors and their corresponding treatment responses by summing z scores from individual treatment-by-interaction test of the five selected genes. Many studies have previously reviewed the theoretical justification of creating such a composite variable and applied the method to combine multiple gene features^25,29–31^. The resultant MINERVA scores of all the 171 tumors ranged from −7.09 to 2.88 with lower score representing better response to adjuvant TKI. Of note, this composite score alone also significantly interacted with treatment (*P* = 4.29 × 10^−6), indicating its role as a stronger predictor of adjuvant treatment than any individual markers. To further stratify patient benefits, the genomic makeup behind each score and optimum separation of survival were considered. First, 81 tumors (47.4%) that did not carry any alterations in the predictive genes (score = 0) were grouped together. We also included 6 patients with both *NKX2-1* and *RB1* alterations in this group, who were scored 0.16. Under the gene-by-treatment test, *NKX2-1* (z-score, −2.72) and *RB1* (z-score, 2.88) were the strongest predictors for adjuvant therapies but in opposite directions (Table 1). By slightly relaxing the cutoff to include these six patients, we tolerated potential noise of the interaction statistics introduced by the current cohort size. Further, to stratify patients for particular treatment benefits, we evaluated cutoffs of MINERVA score at ±1, ±0.5, and 0 and chose to categorize the patients into three subgroups at −0.5 and 0.5 as they resulted in the best survival differences (Supplementary Fig. 4b and Methods). In the pre-categorized population, gefitinib significantly prolonged the median DFS, and increased the 2-year DFS rate, similar to the intention-to-treat (ITT) and modified ITT populations^6^ (Fig. 2a). Remarkably, after categorization by MINERVA, the three subgroups demonstrated distinct treatment responses and underlying molecular profiles (Fig. 2b, c). The Highly TKI-Preferable group [HTP, *N* = 60, 35% (score ≤ −0.5)] expressed significant superiority with adjuvant gefitinib [HR 0.21 (95% CI 0.10–0.44)], and was enriched with copy number gain of *NKX2-1, CDK4,* and *MYC*, and *TP53* exon 4/5 missense mutations. The TKI-Preferable group [TP, *N* = 87, 51% (score −0.5 to 0.5)] showed improved DFS among the pre-categorized and ITT populations [HR 0.61 (95% CI 0.35–1.07)]. Besides, this subgroup was characterized by the absence of most predictive biomarkers, except for sporadic co-existence of *NKX2-1* and *RB1* alterations, with contrasting effects due to opposing iHRs (Table 1). Moreover, a small subset of patients, the Chemo-Preferable Group [CP, *N* = 24, 14% (score ≥ 0.5)], despite having *EGFR-*positive tumors, showed greater response and enhanced DFS [HR 3.06 (95% CI 0.99–9.53)] under VP treatment, and harbored *RB1* alterations (Fig. 2c).

In the TP group, the Kaplan–Meier estimate depicted similar curvatures as those observed in the pre-stratified and ITT populations^6^ (Fig. 2a, e), indicating that adjuvant gefitinib achieved a superior DFS. Importantly, the survival curves of the post-categorized HTP and CP populations did not converge at any point (Fig. 2d, f). In HTP, the Kaplan–Meier curves separated widely as early as six months, with a slow descent of the adjuvant gefitinib arm (median DFS, 34.5 months; *P* < 0.001). Conversely, a drastic drop of the VP arm towards a median DFS of 9.1 months was observed with all recurrence by 36 months. Therefore, the relative benefit of gefitinib was represented by a 6.4-fold increase in the 2-year DFS rate [70.3% (95% CI, 55.8–88.7) vs 11.0% (3.1–38.7)] and a 25.4-month longer median DFS (Fig. 2d). In the CP group, Kaplan–Meier curves diverged at 18 months with an immediate sharp decline of the gefitinib arm towards a median of 19.3 months. Meanwhile, 70% of the VP arm continued to benefit after 24 months (median DFS, 34.2 months, *P* = 0.041). The superiority of adjuvant VP was reflected by a 1.7-fold increase in the 2-year DFS rate [69.2% (48.2–99.5)], including a 14.9-month longer median DFS, compared to the 41.6% 2-year DFS rate for gefitinib (95% CI 19.9–86.8) (Fig. 2f).

### Stratification of overall survival benefit by MINERVA score

Overall survival (OS) is generally considered as the standard endpoint for clinical trials. Although adjuvant gefitinib has shown significantly improved DFS relative to adjuvant VP, the DFS benefits in the ITT population did not translate into a significant difference in OS of the ADJUVANT trial^32^, probably due to the combined influences of downstream treatment crossovers and the genetic heterogeneity among the patient population. Hence, we further used MINERVA in an attempt to achieve stratification of OS.

**Fig. 3:**
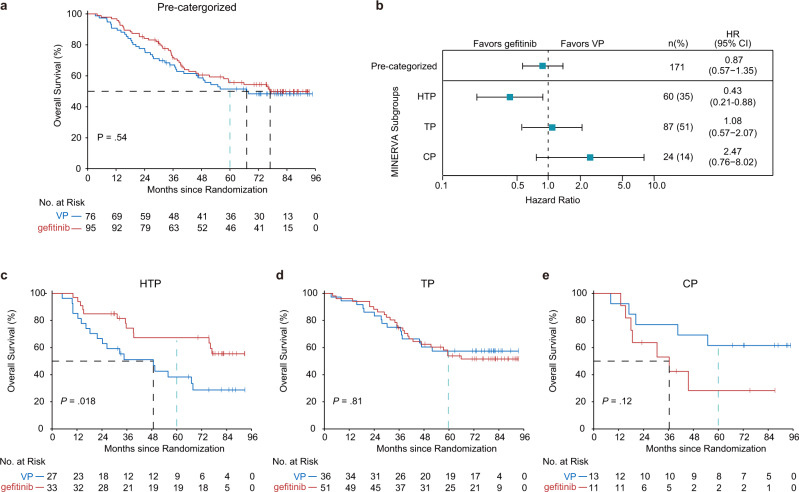
Overall survival (OS) benefit stratification by MINERVA. **a** Kaplan–Meier estimates of OS for the pre-categorized cohort included in this study (*N* = 171). Two-sided *P* value was calculated using log-rank test. **b** Forest plot showing hazard ratio (HR) of OS in MINERVA subgroups. Error bars indicate 95% confidence intervals. **c**–**e** Kaplan-Meier curves estimate OS in each subgroup by treatments. Dotted lines in black indicate median OS. Dotted lines in blue indicate 5-year survival rate (60 months). Two-sided *P* values were derived from the log-rank test.

As expected, OS of the 171 pre-categorized patients involved in this study showed no difference between the two treatment groups (median, 76.9 months in the gefitinib group vs 67.1 months in the VP group; HR 0.87 (95% CI 0.57–1.35), *P* = 0.54) (Fig. 3a and Supplementary Fig. 5). Promisingly, MINERVA successfully demonstrated the stratification of OS benefit as well. In HTP, gefitinib treatment led to significantly longer OS [median, not reached in the gefitinib group vs 48.7 months in the VP group; HR 0.43 (95% CI 0.21–0.88), *P* = 0.018] with a clear and early separation of the Kaplan–Meier curves (Fig. 3b, c). Conversely, adjuvant VP treatment substantially improved OS in the CP group after 18 months [median, 36.4 months in the gefitinib group vs not reached in the VP group; HR 2.47 (95% CI 0.76–8.02), *P* = 0.12] (Fig. 3b, e). OS in TP mirrored that of the pre- categorized cohort, suggesting no differences between the treatments (Fig. 3a, d). Likewise, the 2-, 3- and 5-year survival rates of the categorized subgroups demonstrated similar trends, with the survival differences between the two treatments in both HTP and CP groups widened over time (Supplementary Fig. 6). The 5-year OS rates of gefitinib-treated HTP patients and VP-treated CP patients were 67.3% (95% CI 52.4–86.4) and 61.5% (95% CI 40.0–94.6), respectively, both of which were significantly higher than those attained in the pre-categorized cohort [gefitinib, 55.7% (95% CI 46.2–67.0); VP, 51.5% (95% CI 41.2–64.3)].

### Validation of MINERVA score

**Fig. 4:**
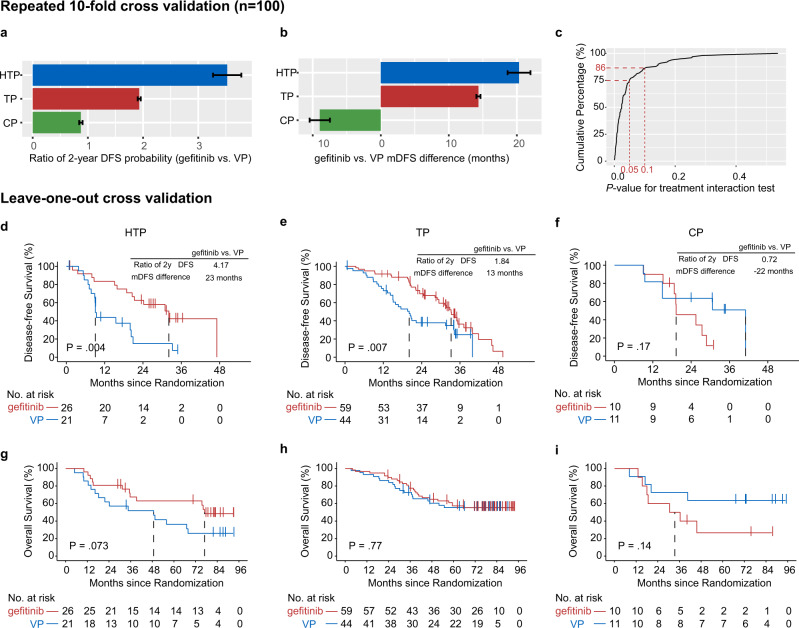
Internal validation of MINERVA. **a**, **b** Ten-fold cross validation was repeated 100 times to assess relative benefit among three MINERVA risk groups by (**a**) mean ratio of 2-year disease-free survival (DFS) probability comparing gefitinib to VP from 100 repeats, and (**b**) mean difference in median DFS between gefitinib and VP treated patients from 100 repeats. Error bars indicate standard error of 100 repeats in each subgroup. **c** Curve showing the cumulative percentage of mock MINERVA models from 100 repeated 10-fold cross validation and corresponding p-values derived from the MINERVA-by-treatment interaction tests. Red dotted lines indicate percentage of repeats with interaction *P* < 0.05 or <0.1 (two-sided, wald test). **d**–**f** Kaplan-Meier estimates of DFS in three mock MINERVA subgroups derived by leave-one-out cross validation. *P* values were derived from the two-sided log-rank test. **g**–**i** Kaplan-Meier estimates of OS in three mock MINERVA subgroups. *P* values were derived from the two-sided log-rank test. Source data used to generate this figure are provided as a Source Data file.

We employed both ten-fold cross validation as well as LOOCV methods (as internal validation procedures) to evaluate the robustness of our MINERVA score. For each fold of cross validation, a subset of markers was selected based on interaction *P* < 0.05, which were then used to build mock MINERVA scores for internal validation. A relatively superior survival with adjuvant gefitinib treatment was observed in both HTP and TP subgroups, with an average of 3.5- and 1.9-fold increase in the 2-year DFS rate, respectively (Fig. 4a). The median DFS in these two subsets also increased by an average of 20 and 15 months, respectively (Fig. 4b), while the 2-year gefitinib-to-VP DFS ratio was less than one, and the median DFS difference negative for all repeats in the CP group, suggesting greater survival benefit by adjuvant VP in this population. Among the 100 mock MINERVA score generated, 75% demonstrated significant treatment interaction with *P* values < 0.05, while 86% demonstrated interaction *P* values < 0.1 (Fig. 4c). We further validated the functionality of the original MINERVA score by LOOCV method. Adjuvant VP treatment in the HTP group was associated with markedly reduced DFS and OS (Fig. 4d, g). Meanwhile, adjuvant gefitinib treatment in the CP group was evidently inferior, similar to previously estimated results in Figs. 2 and 3.

We further validated MINERVA in an independent cohort with similar clinical context (*EGFR*-mutant, Stage IIIA-N2 NSCLC) (see Methods). We performed the same NGS profiling of twenty-nine patients from the EMERGING-CTONG1103 trial recruited in our center (Guangdong Provincial People’s Hospital) and scored them according to MINERVA. Importantly, treatment interaction test indicated that the MINERVA score alone was a strong predictive biomarker to guide treatment selection in both exploratory and validation cohorts (ADJUVANT cohort, *P* = 4.29 × 10^−6; EMERGING cohort, *P* = 0.00032) (Supplementary Fig. 7b). Patients were then classified into HTP, TP and CP groups using score cutoffs at 0.5 and −0.5. We observed similar stratification of patient outcomes as the described above (Supplementary Fig. 7c–e). Specifically, TKI-treated patients showed significantly better progression-free survival (PFS) than the chemotherapy-treated in the HTP group (*P* = 0.039; erlotinib, median PFS 23.8 months, chemotherapy, median PFS 4.5 months) (Supplementary Fig. 7c). Also, comparable PFS benefit was seen with erlotinib in the TP group, in the validation cohort and the entire EMERGING population (Supplementary Fig. 7a, d)^33^. In the CP group, we observed shorter PFS with TKI (median PFS 11.8 months vs 17.7 months TKI-treated in the whole validation cohort) and potential sensitivity to chemotherapy despite the limited sample size (Supplementary Fig. 7e).

## Discussion

To date, several prospective clinical trials, including our ADJUVANT trial, have presented the superiority of adjuvant TKI in early-stage *EGFR*-mutant NSCLC. The ADJUVANT trial had reached a median OS of 75.5 months by the database lock date, which is one of the best survival outcomes ever recorded for this patient population^34^. However, gefitinib’s DFS superiority started declining after 36 months, and did not ultimately translate into OS benefit, raising concerns over achieving clinical cure by adjuvant TKI^35^. The heterogeneity in time-to-recurrence and OS observed within the ADJUVANT cohorts suggested high inter-tumor molecular heterogeneity in early-stage *EGFR*-mutant NSCLC^11^, necessitating additional predictive biomarkers to redefine personalized adjuvant therapy.

In this biomarker exploration of adjuvant TKI in resected NSCLC, we selected five genes that could independently predict the relative benefit between adjuvant TKI and chemotherapy. The multigene MINERVA score then integrated these biomarkers and effectively compensated for individuals’ molecular heterogeneity. Notably, the three risk groups separated using this score counteracted the controversial impermanence of DFS benefit with exciting stratification of OS benefits as well. For each risk groups, we also found characteristic enrichment of biomarkers, possibly explaining their differential responses to adjuvant treatments.

In the CP group, *RB1*-altered/*EGFR*-mutant patients showed better survival with adjuvant VP rather than gefitinib. In consistent with our observation, a number of studies have reported particularly poor *EGFR-*TKI response in *RB1* altered patients. For example, Kim et al. reported a significantly shorter median PFS of only 1.9 months in *RB1*-mutant patients in contrast to 11.7 months in *RB1* wildtype patients^36^. In advanced NSCLC, TKI-resistant *RB1*-inactivated/*EGFR*-mutated adenocarcinoma clones have been found to transdifferentiate into small-cell lung cancer (SCLC) and become more responsive to chemotherapy^37,38^. One of the potential mechanisms of SCLC transformation might be the disruption in expression of cell-state-determining factors due to *RB1* inactivation^15,39^. The resulting lineage plasticity then converts the therapy-dependent cancer cells to those that express neuroendocrine lineage markers, making them refractory to targeted treatments^40,41^. *RB1* often demonstrates mutual exclusivity with cell cycle pathway genes, and reflects chemosensitivity in rapidly progressing tumors^42^, which is in line with our CP population. In hope to eradicate this subclone, researchers have developed an upfront trial in which patients with advanced stage were assigned to receive TKI and small-cell directed chemotherapy (platinum plus etoposide) alternately (NCT03567642). Further research is required to explore whether TKI insensitivity of RB1-inactive/EGFR-mutant patients in the adjuvant setting also arise from early histological transformation events.

Despite the small VP-favoring population, patients in the HTP subgroup presented significant benefits from adjuvant gefitinib therapy. Genomic analysis showed the enrichment of other four biomarkers, among which copy number gain of *NKX2-1* received nearly equal weightage as *RB1*, but in an opposite predictive direction that favors the choice of gefitinib. *NKX2-1* copy number gain is a widely prevalent oncogene found in up to 30% of *EGFR*-mutant patients^10^. *NKX2-1* amplification has been more frequently observed in TKI-treated patients with extended progression-free survival (≥24 months) and was reported to predict favorable TKI response^14,43^. These findings were consistent with its enrichment in the HTP population with relative gefitinib benefit. Previous studies mainly reported favorable prognosis with *NKX2-1* expression in mixed onco-driver, tumor stages and pathological backgrounds^44,45^, while our study demonstrated the predictive value of *NKX2-1* copy number gain to favor adjuvant TKI treatment in a more defined population.

*TP53* as a tumor suppressor occurred in more than 50% of NSCLC, with mutations of complicated functional properties^16^. Unlike most tumor suppressor genes, missense mutations in the critical DNA binding domain (exons 4 to 8) are the most common variants in *TP53*, which are associated with the lower disease control rate and poorer survival outcomes with TKI treatment in contrast to *TP53* wildtypes in *EGFR*-mutant NSCLC^16,46^. Apart from poor prognosis associated with loss-of-function *TP53* mutations, studies have also revealed varied prognostic effects of missense mutations on different *TP53* exons, suggesting possible functional divergence^28,47–50^. Interestingly, these missense variants could also be predictive for worse adjuvant chemotherapy outcomes compared to observation in resected NSCLC^51^. Along these lines, in this study, the predictive power of *TP53* variants was also assessed by exons. In the multigene model, the positive predictivity of *TP53* exons 4/5 missense mutations suggested that patients harboring these *TP53* mutations would relatively benefit more from gefitinib than VP. We did not consider exons 6–8 because they lacked predictive significance for adjuvant therapies under the treatment-interaction test. In concert with the consensus on *TP53*’s negative prognostic effect, we did observe significantly lesser outcomes in *TP53*-positive patients despite the treatment they received. *MYC* amplification was found in approximately 10% NSCLC^10^. Here, we demonstrated that it was predictive for better outcome with adjuvant TKI than chemotherapy. In consistent with our finding, a previous report also showed that *MYC* amplification was associated with better response to gefitinib^52^. On the other hand, amplification and overexpression of MYC was often found to correlate with chemoresistance in lung cancers^53^. CDK4 also frequently overexpressed in lung cancers^54^, although it was not found to particularly affect gefitinib outcome according to a thorough literature search, as demonstrated by an indifferent efficacy (*p* = 0.813) in patients with or without *CDK4* gain^55^. CDK4 was a key member of the cyclin-dependent kinase family that phosphorylates RB1 and facilities the cell cycle activities. It has been reported in many other cancers that *CDK4* amplification could drive resistance to chemotherapy, including osteosarcoma and breast cancer^56,57^. Its role in predicting better adjuvant TKI response than chemotherapy might be worthy of future investigation.

The development of a multi-gene clinical predictor requires a well-designed prospective validation with appropriate assumptions and sample size structured to address the underlying molecular heterogeneity. However, this is challenging as there were no equivalent public or clinical datasets of adjuvant TKI-treated patients with regular follow-up of survival outcome available at the time of this study, and any prospective validation trials might span over another decade to reach maturity. In this regard, we tested the validity of our score in another prospective clinical trial conducted by the Chinese Thoracic Oncology Group (CTONG), the EMERGING-CTONG1103 trial, a multicenter phase II neoadjuvant study that enrolled patients with EGFR-mutant stage IIIA-N2 NSCLC^33^. As neoadjuvant treatment mainly contributed to the overall response rate, patients’ survival benefits were achieved primarily from adjuvant treatment. We considered the EMERGING study to be the most appropriate cohort by now to partly resemble the ADJUVANT dataset. Both cohorts examined *EGFR*-mutant patients in the early-stage context, indicating similar baseline genomic makeup that might influence treatment response^11,58^. Here, we saw similar separation of TKI or chemotherapy benefit and predictive value of the score as a composite variable. Results from this cohort verified the potential generalization of MINERVA-guided treatment strategies in early-stage *EGFR*-mutant patients. Despite inspiring results from this independent validation cohort, we acknowledged the fundamental differences in trial designs. We were cautious when interpreting the results as we cannot completely isolate the influence from neoadjuvant treatment in EMRGING-CTONG1103. Besides, only a small cohort was collected for validation and further validation in larger populations in the adjuvant setting is needed. Due to these concerns, we are providing results of this independent validation in the supplementary data. Moreover, development of this score was based on a relatively small training cohort, which may introduce biased biomarker selection, or an overfitted model. Therefore, it is important to exploit stringent statistical procedures to minimize cherry-picking during post hoc analyses. Cross-validating all the steps of model construction allowed us to evaluate whether the current algorithm could be uniformly applied to the entire cohort. Besides, only baseline specimen was examined in our study, while dynamic minimal residual disease (MRD) detection might provide additional information for the application of precise adjuvant TKI. However, consensus opinion on MRD’s definition and detection technologies needed to be settled first.

Other limitations include insufficient tissue availability for retrospective genomic analysis of all the enrolled participants. However, both clinical characteristics and survival outcomes of the pre-categorized patients in this study were matched with those of the ITT population from the ADJUVANT trial.

In summary, this exploratory retrospective analysis of the ADJUVANT trial has unraveled the genetic constructs of *EGFR* co-mutations in stage II and III resected NSCLC. Further, the interplay between identified predictive markers and clinical outcomes has been carefully examined, and incorporated into a multi-gene predictive score to aid the adjuvant paradigm. Our evidential MINERVA score presents a fresh perspective for future studies to examine its clinical validity, thereby guiding the development of more personalized adjuvant therapies, and their transition from bench to bedside.

## Methods

### Adjuvant treated patients

All patients had Stage II–IIIA (N1-N2), *EGFR-*mutant NSCLC, underwent complete surgical resection and were randomized in the ADJUVANT/CTONG1104 trial (NCT01405079)^6^. All except 27 patients initiated either adjuvant gefitinib or intravenous vinorelbine plus cisplatin between September 2011 and April 2014. The independent validation cohort included patients from the EMERGING-CTONG1103 trial, which was a multicenter phase II neoadjuvant study of patients with previously untreated EGFR-mutant stage IIIA-N2 NSCLC. Total 72 patients were randomly assigned between December 5, 2011, and December 13, 2017 to receive neoadjuvant erlotinib for 42 days or two cycles of gemcitabine/cisplatin and up to 12 months of adjuvant erlotinib or chemotherapy after surgery (one withdrew before treatment). For both clinical trials, *EGFR*-activating mutations were evaluated using amplification-refractory mutation system PCR at time of enrollment. All patients provided written informed consent for participating in the ADJUVANT/CTONG1104 or the EMERGING-CTONG1103 trial, and this predefined biomarker study was included in the consent forms for both studies.

### Clinical efficacy assessment

Per protocol, patients were assessed for DFS by chest CT scan and abdominal ultrasound every 3 months, brain MRI every 6 months, bone scan every 12 months from baseline until disease relapse or death (whichever comes first) for up to 3 years. The survival after 3 years will be followed up semi-annually with telephone. Secondary endpoints, including OS, 3-year DFS rate, 5-year DFS rate, and 5-year OS rate, were also evaluated. Patients who were alive or lost to follow-up were censored on the last day they confirmed survival. The baseline demographics, clinical characteristics, and the primary end point data of ADJUVANT-CTONG1104, including the DFS, were collected from the previous publication^6^. OS was updated either by phone interview, or on-site follow-up of the enrolled patients^32^. For independent validation cohort, clinical data, including PFS and OS, were obtained from our previous publication of EMERGING-CTONG1103 and patients underwent the same schedule of long-term follow-up as the ADJUVANT trial^33^. The ADJUVANT-CTONG1104 trial was approved by the research ethics boards of Guangdong Provincial People’s Hospital and all other participating hospitals (including Fudan University Affiliated Zhongshan Hospital, Shanghai, China; Zhejiang Cancer Hospital, Hangzhou, China; Hunan Cancer Hospital, Changsha, China; The Affiliated Hospital of Medical College Qingdao University, Qingdao, China; Liaoning Cancer Hospital, Shenyang, China; Fujian Medical University Union Hospital, Fuzhou, China; Jilin Provincial Tumor Hospital, Changchun, China; Jiangsu Cancer Hospital, Nanjing, China; The People’s Hospital of Peking University, Beijing, China; Shanghai Pulmonary Hospital, Tongji University, Shanghai, China; Tangdu Hospital, Xi’an, China; Peking University First Hospital, Beijing, China; Fujian Cancer Hospital, Fuzhou, China; Beijing Chest Hospital, Beijing, China; The First Hospital of China Medical University, Shenyang, China; Beijing Cancer Hospital, Beijing, China; Harbin Medical University Cancer Hospital, Harbin, China; West China Hospital of Sichuan University, Chengdu, China; Sichuan Cancer Hospital, Chengdu, China; The Northern Jiangsu People’s Hospital, Yangzhou, China; The First Affiliated Hospital of Suzhou University, Suzhou, China), and the EMERGING-CTONG1103 trial was approved by the research ethics boards of Guangdong Provincial People’s Hospital and participating hospitals (Peking University Cancer Hospital and Institute, Beijing, China; Fujian Medical University Union Hospital, Fuzhou, China; First Affiliated Hospital of Dalian Medical University, Dalian, China; Peking University People’s Hospital, Beijing, China; Zhejiang Cancer Hospital, Hangzhou, China; Zhongshan Hospital, Shanghai, China; Guangzhou Liuhuaqiao Hospital, Guangzhou, China; Jilin Provincial Tumor Hospital, Changchun, China; Jiangsu Cancer Institute and Hospital, Nanjing, China; Tianjin Medical University Cancer Institute and Hospital, Tianjin, China; First Affiliated Hospital of Xi’an Jiaotong University, Xi’an, China). Both trials were conducted in accordance with the ethical principles of the Declaration of Helsinki.

### Sample details

From the ADJUVANT/CTONG1104 trial, 175 patients treated by either adjuvant arms had sufficient and qualified archived formalin-fixed paraffin-embedded (FFPE) tumor tissue specimens obtained from 25 centers. A minimum of five FFPE slides or 250 ng genomic DNA was required. Of these, 171 patients positive for *EGFR* by NGS were subjected to further biomarker analyses (including 95 from the gefitinib group and 76 from the VP group). For the independent validation cohort from the EMERGING-CTONG1103 trial, 37 patients were recruited at our center (Guangdong Provincial People’s Hospital), whose archived pre-treatment needle-aspiration FFPE samples we have access to. Of these, 29 patients had enough samples for NGS testing and were therefore included in this biomarker analysis (including 15 from the erlotinib arm and 14 from the chemotherapy arm).

### DNA extraction and sequencing library preparation

Samples were sent to the CAP/CLIA (College of American Pathologists and Clinical Laboratory Improvements Amendments) accredited central laboratory at Nanjing Geneseeq Technology Inc. (Nanjing, China) for genomic DNA extraction and hybridization capture-based targeted NGS of 422 cancer-relevant genes. Protocols from previous publication were followed for both experimental procedures as well as sequence data analysis^59^. In detail, five to eight 10 µm FFPE sections were first de-paraffinized with xylene and then used for genomic DNA (gDNA) extraction by QIAamp DNA FFPE Tissue Kit (Qiagen) following the manufacturer’s instructions. The extracted gDNA samples were quantified on Qubit 3.0 fluorometer (Thermo Fisher Scientific) and its purity was measured on Nanodrop 2000 (Thermo Fisher Scientific), following by fragmentation to a size around 350 bp by using Covaris M220 sonication system (Covaris) and then purified by size selection with Agencourt AMPure XP beads (Beckman Coulter).

Fragmented gDNA were used to prepare DNA libraries with KAPA hyper library preparation kit (KAPA Biosystems) according to the manufacturer’s protocol. Libraries were then subjected to PCR amplification and purification with Agencourt AMPure XP beads before targeted enrichment.

Libraries with different sample indices were first pooled together to a total DNA amount of 2 µg and then subjected for targeted enrichment with IDT xGen Lockdown Reagents and a customized enrichment panel (Integrated DNA Technologies) covering the exonic regions of 422 genes and the introns of 16 fusion genes. The captured library was further amplified with Illumina p5 (5ʹ AAT GAT ACG GCG ACC ACC GA 3ʹ) and p7 (5ʹ CAA GCA GAA GAC GGC ATA CGA GAT 3ʹ) primers in KAPA Hifi HotStart ReadyMix (KAPA Biosystems, Wilmington, MA) and purified with Agencourt AMPure XP beads. Sequencing libraries were quantified by qPCR with KAPA Library Quantification kit (KAPA Biosystems) and its size distribution was examined on Bioanalyzer 2100 (Agilent Technologies). The final libraries were sequenced on Illumina Hiseq 4000 platform for 150 bp paired-end sequencing according to the manufacturer’s instructions. All experimental procedures were performed using validated assays.

### Sequencing data analysis

Raw sequencing data was analyzed by a validated automation pipeline. In brief, raw data were first subjected to bcl2fastq for demultiplexing and then Trimmomatic^60^ for FASTQ file quality control to remove low quality (base phred score below 30) and N bases. Reads were aligned to the reference human genome hg19 by Burrows-Wheller Aligner (BWA-mem, v0.7.12)^61^. PCR duplicates were removed by Picard. Genome Analysis Toolkit (GATK 3.4.0) was employed to apply the local realignment around indels and recalibrate base quality score. Single-nucleotide variations (SNVs) and insertion/deletion mutations were called using VarScan2 with the following parameters: (1) for mutations with more than 20 recurrences in COSMIC, minimum variant allele frequency (VAF) = 0.01 with at least three minimum variant supporting reads; (2) for others, minimum VAF = 0.02 with at least five minimum variant supporting reads; in addition, all variants also need to meet the standards of minimum read depth = 20, minimum base quality = 25, variant supporting reads mapped to both strands, and strand bias no greater than 10%.

CNV detection was using a self-developed pipeline, which has been validated in 38 samples against their droplet digital polymerase chain reaction (ddPCR) results as “gold standard”. The system noise in copy number data was reduced by principal component analysis of 100 normal samples sequenced in the same batch.

### Variant filtering and annotation

Single-nucleotide polymorphism (SNPs) and small insertions/deletions (indels) were annotated by ANNOVAR against the following databases: dbSNP (v138), 1000Genome, ExAC, COSMIC (v70), ClinVAR, and SIFT. Mutations that were presented in >1% population frequency in the 1000 Genomes Project or 65000 exomes project (ExAC) were removed. The resulted mutation lists were filtered through an internally collected list of recurrent sequencing errors on the same sequencing platform, which is summarized from the sequencing results of 200 normal samples with a minimum average sequencing depth of 700×. Specifically, if a variant was detected (i.e. ≥3 mutant reads and >1% VAF) in >20% of the normal samples, it was considered a likely artifact and was removed. Mutations occurred within the repeat masked regions were also removed.

### Selection of predictive gene features

We followed a well-established method to select predictive biomarkers and develop the composite score^25,29,30^. We first performed an interaction test between treatment and each candidate gene separately. Specifically, for a particular gene feature, we assume a standard multivariate Cox proportional hazards model:

1 $${h}_{i}\left(t\right)={h}_{0}\left(t\right){\exp }\left\{{\psi }_{1}{r}_{i}+{\psi }_{2}{g}_{i}+{\psi }_{3}{r}_{i}{g}_{i}\right\}$$

where *h*_0_ (*t*) is the baseline hazard and $\psi$ are the regression parameters, ${gi}$ the mutation status of the gene feature, *r*_*i*_ denotes the treatment assignment for patient *i*, such that *r*_*i*_ = 0 indicates that the patient received chemotherapy and *r*_*i*_ = 1 indicates that gefitinib was administered, and the product ${r}_{i}{g}_{i}$ represents the interaction term of treatment and mutation status of the gene.

Selection of biomarker is based on the Wald test statistic for testing a null interaction effect, ${\psi }_{3}$= 0. A standardized test statistic, *z*, that approximately follows the standard normal distribution under the null interaction effect was calculated by:

2 $$z=\frac{{iHR}}{{se}({iHR})}$$

where *iHR* stands for interaction-hazard ratio derived from the interaction term of Eq. (1).

After standardization, a negative or positive *z* value represents that alteration in a gene is associated with better outcome with adjuvant gefitinib or TKI, respectively. A set of features with significance level of this test statistics less than 0.05 was used to generate the multi-gene score. All selected markers were also evaluated for whether they were confounded by any clinical variables through multivariate analysis, with the following controlled: age, sex, smoking history, clinical stage, and lymph node stage (N stage). A treatment-adjusted Cox’s model, without the interaction term, was used to test for prognostic biomarkers as a reference that were not involved in the score development.

### Development of the MINERVA score

We adapted a well-recognized linear discriminant model to compile all predictive biomarkers into a composite MINERVA (Multiple-gene INdex to Evaluate the Relative benefit of Various Adjuvant therapies) score to address underlying molecular heterogeneity. This is usually done by summing weighted averaging of individual variables, which could avoid interference from any large variance in one of the original predictors and control Type I error rate for limited sample sizes^25,29–31^. The resultant composite score captures all meaningful information and can be additive for the predictive effect. Here, this was done by summing the standardized z-scores of the original iHRs. The score for each patient was calculated using the following equation:

3 $${{MINERVA}}_{i}=\mathop{\sum}\limits_{g\in G}{z}_{g}{p}_{i,g}$$

where *G* is the set of selected genes $g$, ${z}_{g}$ is the standardized test statistic of the interaction test for gene $g$, *i* is the *i*^th^ patient, while ${p}_{i,g}$ is the mutation status of $g$ in the *i*^th^ patient where ${p}_{i,g}$= 1 if the genetic feature is altered and ${p}_{i,g}$= 0 otherwise. Therefore, based on interaction z-scores of the five predictive markers, we could calculate the score using the following function:

$$\begin{array}{c}{{{{{\rm{MINERVA}}}}}}=2.88* RB1+(-2.72)* {NKX2\mbox{-}1}\,+\\ (-2.26)* CDK4+(-2.11)* TP53+(-1.98)* MYC.\end{array}$$

Smaller values of MINERVA correspond to greater chance of benefiting from TKI than from chemotherapy as an adjuvant treatment. Based on combination of predictive genes of each score and the best separation of survival stratification, the patients could be effectively categorized into three subgroups with scores of <−0.5, −0.5 to 0.5, and >0.5, with distinct DFS outcomes. The relative survival benefit was compared using a 2-year DFS probability ratio and median DFS difference between gefitinib and VP arms^62^.

### Validation of the MINERVA score

Ten-fold cross validation (CV) was used to evaluate the performance of our predictive model. Inside each fold, gene-by-treatment interaction effect was assessed by cox proportional hazard model based on 90% of the samples and predictive markers with interaction p < 0.05 were selected. Mock MINERVA scores were then calculated for the remaining 10% samples using the selected subset of markers. A complete set of scores can be computed through ten repeated 10-fold CV and patients were assigned to one of the three MINERVA subgroups accordingly (HTP <−0.5, TP −0.5–0.5, or CP > 0.5) to compare survival outcomes. Predictive effect of the MINERVA scores as a composite variable was then evaluated with interaction P-value. This process was repeated 100 times. Relative adjuvant treatment benefit within each subgroup was compared using 2-year DFS probability and median DFS difference between gefitinib and VP arms.

Next, we performed leave-one-out cross validation (LOOCV) to obtain individual scores. Each patient was omitted once in alternation and then scored by models constructed on the remaining 170 patients (using the same marker selection criteria). The patient was then assigned to one of subgroups with the same score cutoffs. The entire procedure was repeated until each patient was left out once, scored and assigned. Kaplan-Meier curves were estimated for each subgroup to evaluate relative benefit of adjuvant gefitinib to VP therapy using 2-year DFS ratio, median DFS difference and the log-rank P values.

For independent validation, genomic profiles of twenty-nine patients from the EMERGING were obtained. Each patient was scored according to their makeup of the five MINERVA genes and assigned into HTP, TP or CP as described above. Survival difference of TKI- and chemotherapy-treated patients were compared using Kaplan-Meier estimates, median PFS and log-rank P-values, which were used to assess generalization of the MINERVA score. Treatment-interaction test with MINERVA scores in this cohort was also performed to validate its predictive value.

### Statistical analysis

Univariate and multivariate analyses of the association of biomarkers, treatment interaction, and clinical factors with DFS were performed with the Cox proportional hazard regression model. Kaplan-Meier curves of DFS and OS were estimated for each subgroup, and statistically compared using the log-rank test. A two-sided *P*-value < 0.5 was considered statistically significant. All statistical analyses were performed using R software (version 3.5.0).

### Reporting summary

Further information on research design is available in the Nature Research Reporting Summary linked to this article.

## Supplementary information

Supplementary information

Reporting Summary

## Data Availability

The raw sequencing data generated in this study is deposited in the Genome Sequence Archive in fastq format under the accession link HRA001462 and the variants data are deposited in the European Genome-Phenome Archive (EGA) database in vcf format under the accession link EGAS00001005632. The data are available under restricted access due to data privacy laws. Access can be obtained by contacting corresponding authors of this study at syylwu@live.cn. The processed data matrices have been deposited in GitHub in the repository https://github.com/cancer-oncogenomics/minerva-adjuvant-nsclc. The source data generated from cross validation procedures in this study are provided with this paper. Source data are provided with this paper.

## Source data

Source data file

## Code availability

The R code utilized in this study has been deposited in GitHub in the repository available at https://github.com/cancer-oncogenomics/minerva-adjuvant-nsclc^63^ (10.5281/zenodo.524251).
