# Supplementary material for: Genomic signatures define three subtypes of EGFR-mutant stage II–III non-small-cell lung cancer with distinct adjuvant therapy outcomes
Source: Nat Commun. 2021 Nov 8;12:6450. doi: 10.1038/s41467-021-26806-7 (PMC8575965; doi:10.1038/s41467-021-26806-7)
Supplement: Supplementary file 1 — Supplementary information [file 41467_2021_26806_MOESM1_ESM.pdf]

Supplementary Figure 1. Mutational landscape of all available samples from ADJUVANT-CTONG1104 by Targeted NGS. Only genes that have ≥ 5% mutation frequency in both VP and gefitinib treatment groups were displayed.

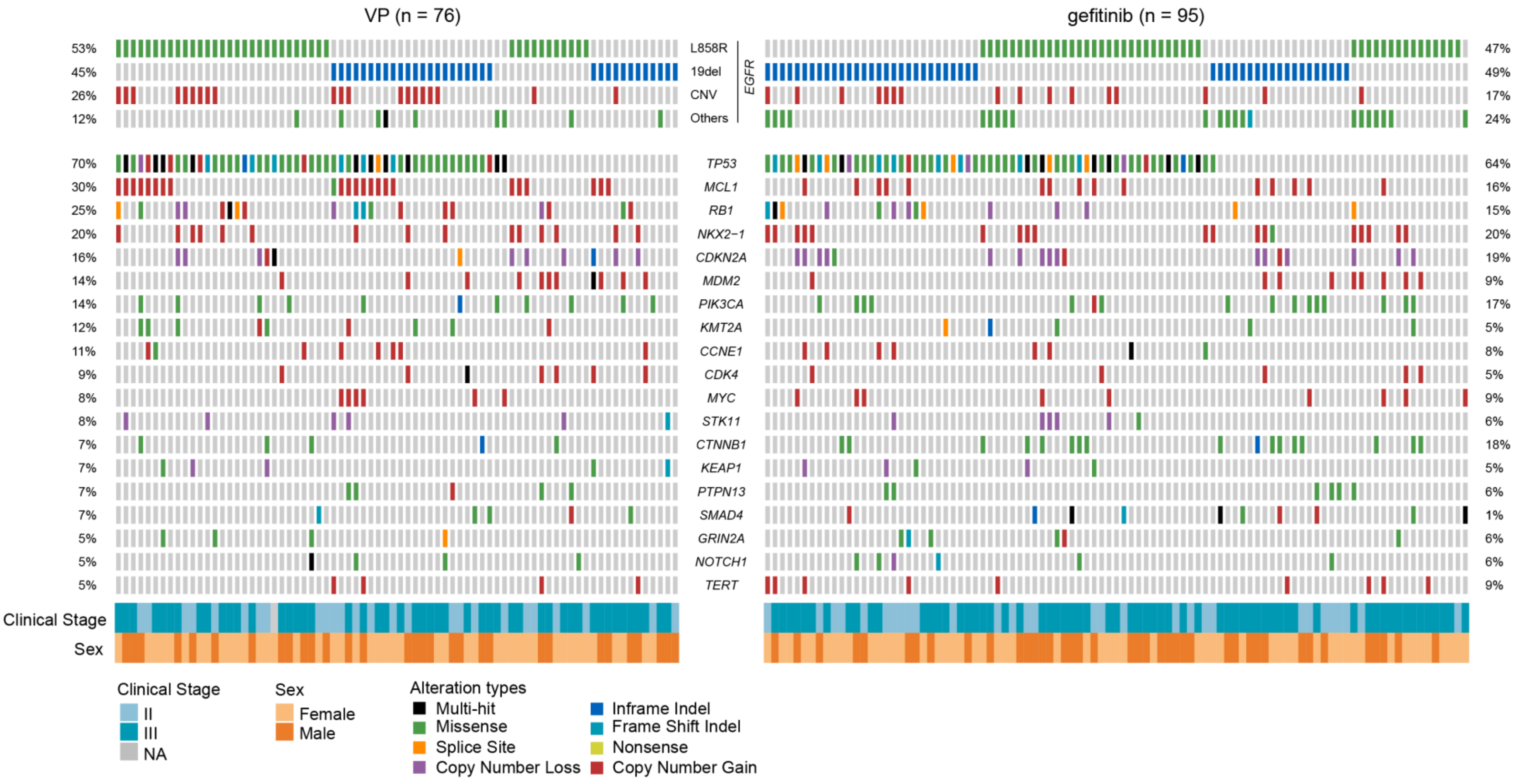

Supplementary Figure 2. Schematic diagram of predictive and prognostic biomarkers for adjuvant TKI and chemotherapy

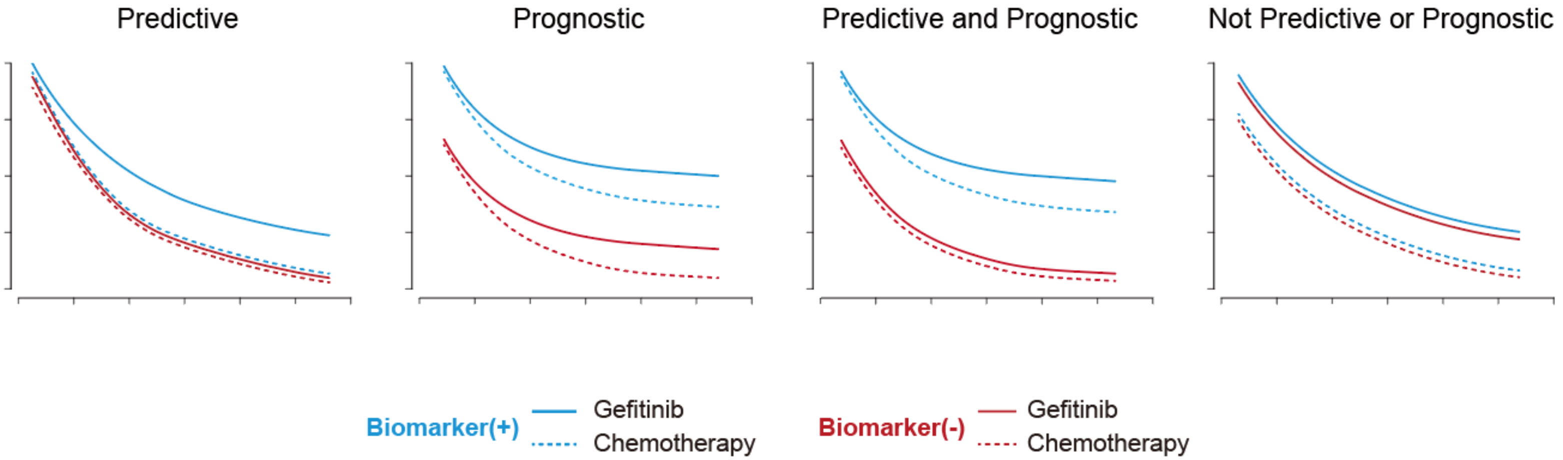

Supplementary Figure 3. Prognostic markers in the exploratory cohort from the ADJUVANT/CTONG1104 study

Kaplan-Meier survival plots showing a) *TP53* exons 4-5 missense mutations, b) *TP53* frameshift and truncating mutations, c) *PIK3CA* mutations, d) *NOTCH1* mutations, and e) *MYC* copy number gain (CNV) positive or negative populations in gefitinib or VP treated arms.

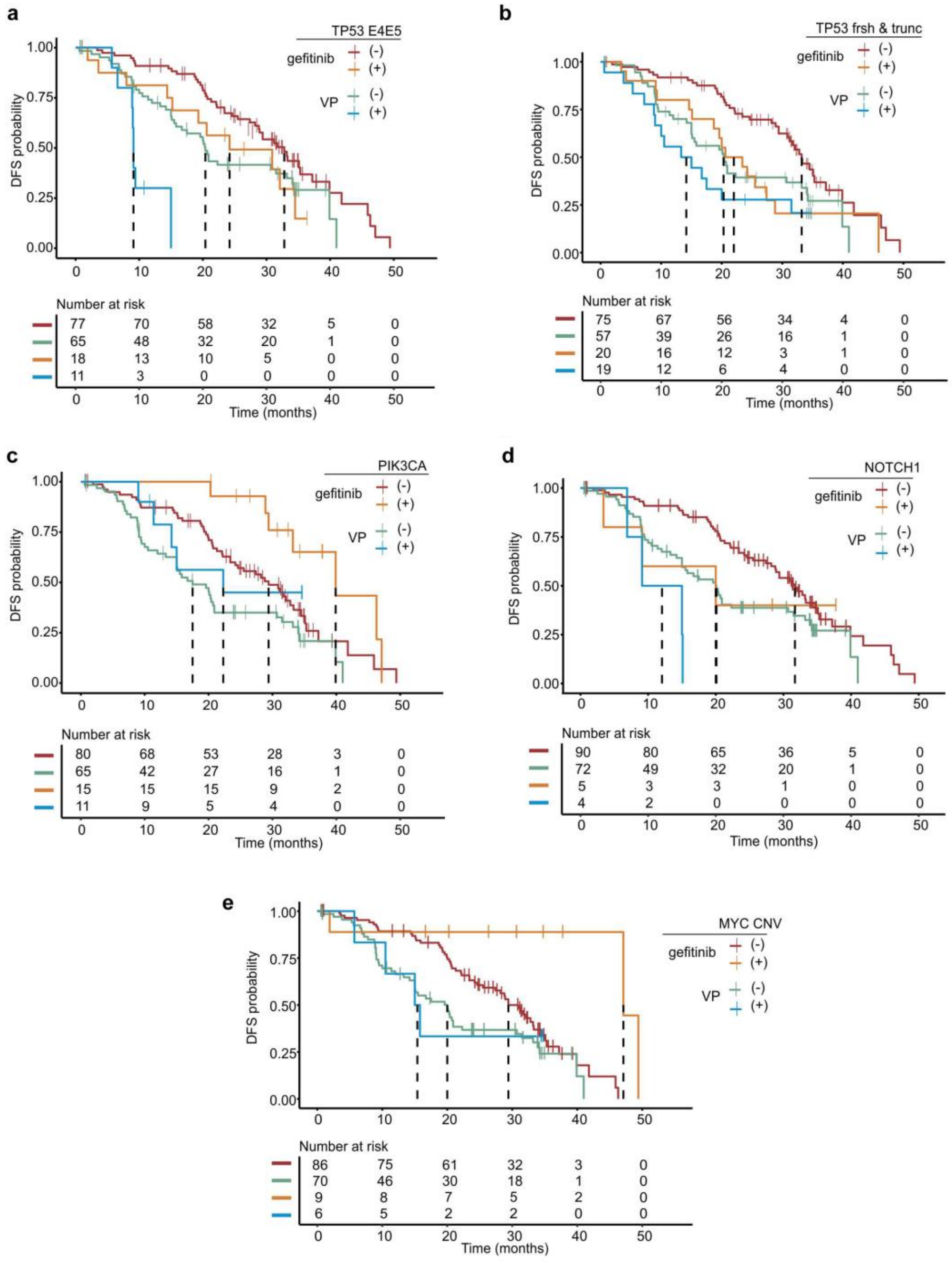

Supplementary Figure 4. Three subgroups identified from MINERVA.
a) Histogram shows the distribution of MINERVA score in entire cohort. b) Comparison of survival differences under different cutoffs of MINERVA. P values are derived from two-sided wald test of the cox proportional hazards regression model.

a

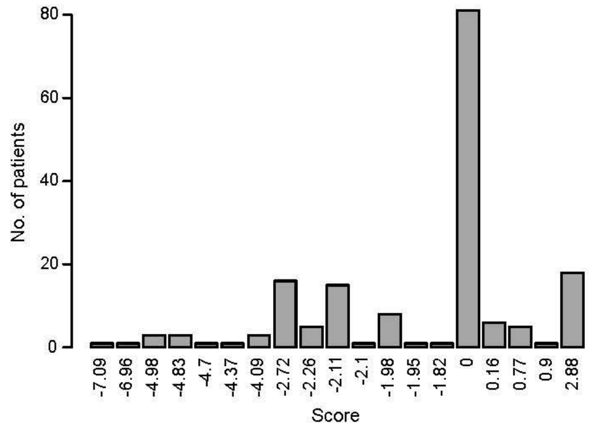

b

cutoffs at	HTP	TP	CP
**-0.5 and 0.5**	n = 60<br>0.21 (0.1-0.44)<br>$p < 2.47 \times 10^{-5}$	n = 87<br>0.61 (0.35-1.07)<br>p = 0.083	n = 24<br>3.06 (0.99-9.53)<br>p = 0.041
**0**	Same as above	n = 81<br>0.65 (0.36-1.16)<br>p = 0.15	n = 30<br>2.26 (0.87-5.88)<br>p = 0.084
**-1 and 1**	Same as above	n = 93<br>0.66 (0.39-1.12)<br>p = 0.12	n = 18<br>3.95 (0.94-16.56)<br>p = 0.06

Supplementary Figure 5. Analysis of overall survival by prespecified subgroups in the exploratory cohort from ADJUVANT-CTONG1104. Forest plot showing the hazard ratio of overall survival between adjuvant gefitinib and vinorelbine/cisplatin chemotherapy (VP) using the cox proportional hazards regression model in prespecified patient subgroups. Error bars indicating 95% confidence intervals of hazard ratios. P values are derived from two-sided wald test of the cox proportional hazards regression model.

	Total number of patients/events (n/N)	Gefitinib (n/N)	VP (n/N)	HR (95% CI)	p value
Male	71/35	40/19	31/16	0.81 (0.42~1.58)	0.54
Female	100/47	55/25	45/22	0.92 (0.52~1.63)	0.77
Smoker	43/22	25/13	18/9	0.91 (0.39~2.13)	0.83
Nonsmoker	128/60	70/31	58/29	0.85 (0.51~1.41)	0.53
EGFR exon19 deletion	93/42	54/23	39/19	0.85 (0.46~1.57)	0.61
EGFR exon21 L858R	78/40	41/21	37/19	0.91 (0.49~1.69)	0.76
Adenocarcinoma	163/77	89/40	74/37	0.84 (0.54~1.32)	0.46
Nonadenocarcinoma	8/5	6/4	2/1	1.44 (0.16~13)	0.75
N1	57/20	31/10	26/10	0.78 (0.33~1.88)	0.58
N2	113/61	64/34	49/27	0.91 (0.56~1.52)	0.74

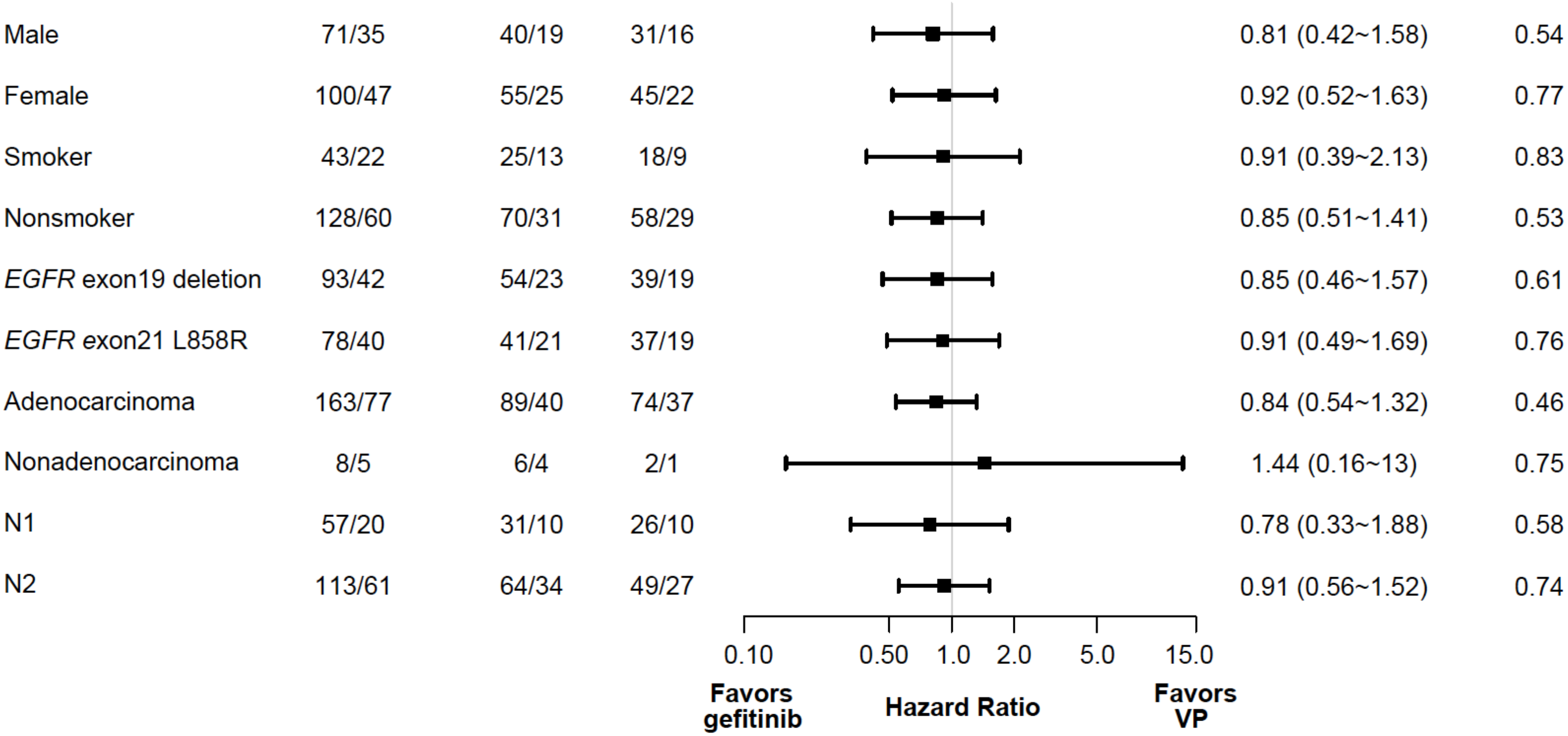

Supplementary Figure 6. Two-year, three-year and five-year overall survival rates in MINERVA stratified patients.
Bar graphs illustrate two-, three- and five-year overall survival rate of the entire pre-stratified cohort (n=171) and three MINERVA score groups (HTP, n=60; TP, n=87; CP, n=24). Error bars indicate 95% confidence interval of survival rates.

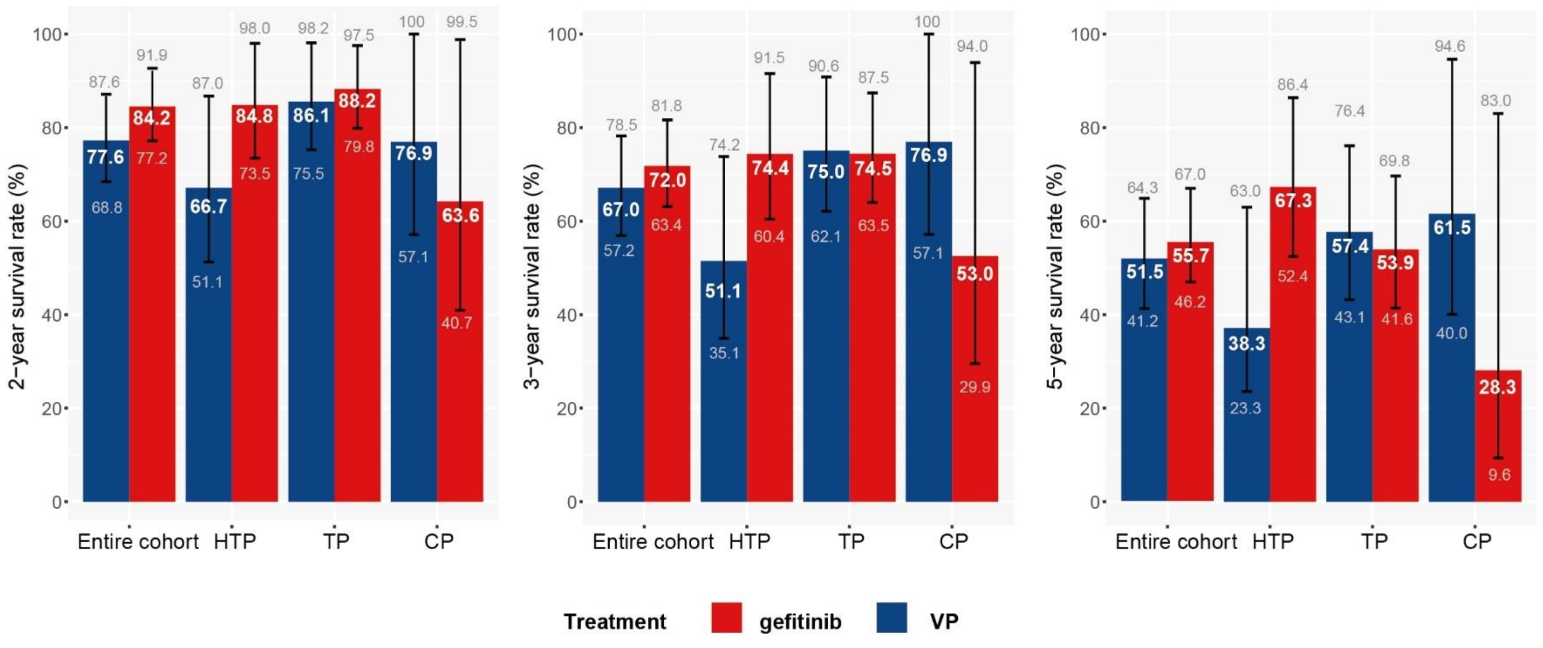

Supplementary Figure 7. Validation of MINERVA in an independent cohort of 29 patients from the EMERGING-CTONG1103 trial. a) Kaplan-Meier curve demonstrating survival of all 29 patients with two-side P value obtained from log-rank test; b) Treatment interaction of MINERVA scores in as a composite variable in ADJUVANT and EMERGING cohorts. P values are derived from two-sided wald test of the cox proportional hazards regression model. c-e) Kaplan-Meier curves of PFS for patients treated by neoadjuvant erlotinib or chemotherapy in three MINERVA subgroups in the independent validation cohort. P values are calculated using two-sided log-rank test.

a

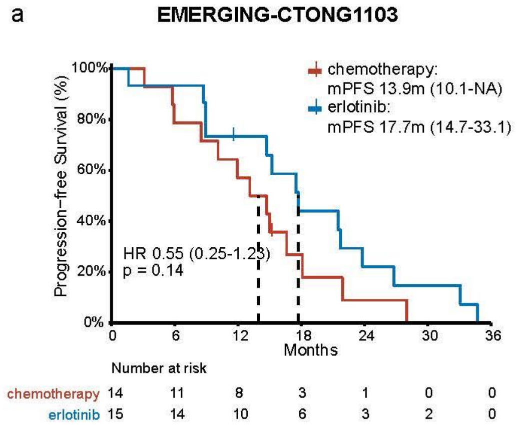

b

	ADJUVANT-CTONG1104		EMERGING-CTONG1103	
	**HR**	**P-value**	**HR**	**P-value**
**Score*Treatment**	**1.64**	**$4.29x10^{-6}$**	**4.61**	**0.00032**
Score	0.72	$1.9x10^{-5}$	0.34	0.004
Treatment	0.62	0.027	0.56	0.19

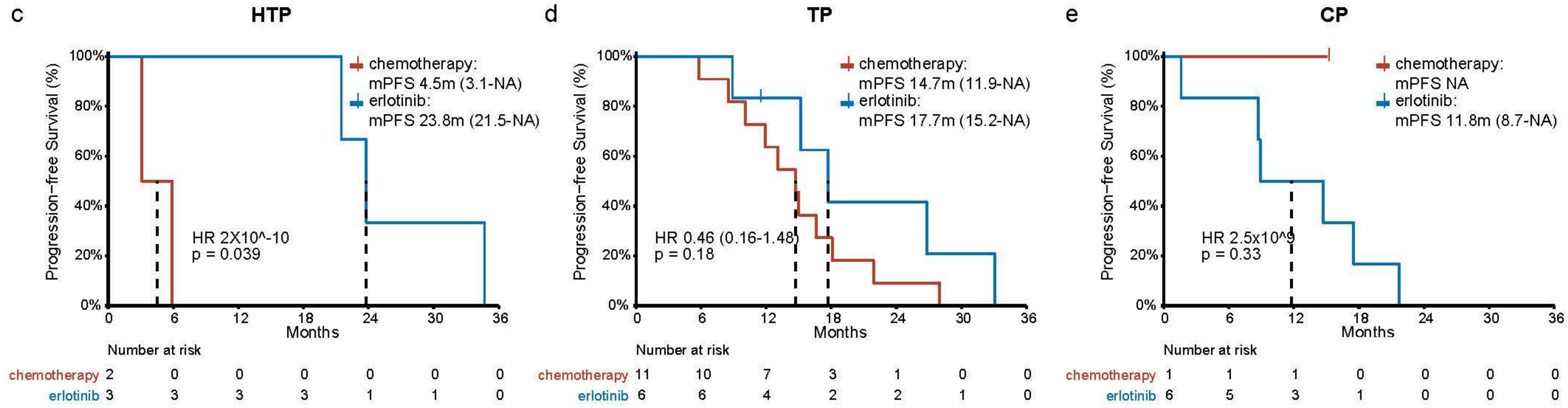

Supplementary Table 1. Summary of basic characteristics in the exploratory cohort from ADJUVANT-CTONG1104

	Exploratory cohort	
	Gefitinib (n=95)	Vinorelbine plus cisplatin (n=76)
**Age (years)**	58 (32-74)	59 (26-73)
**Sex**		
Female	55 (58%)	45 (59%)
Male	40 (42%)	31 (41%)
**Never smoker**	70 (74%)	58 (76%)
**ECOG**		
0-1	93 (98%)	76 (100%)
Not available	2 (2%)	0
**Pathology stage**		
IIA	27 (28%)	24 (32%)
IIB	4 (4%)	2 (3%)
IIIA	65 (68%)	49 (64%)
Not available	0	1 (1%)
**Lymph node status**		
N1	31 (33%)	26 (34%)
N2	64 (67%)	49 (65%)
Not available	0	1 (1%)
**Pathology**		
Adenocarcinoma	89 (93%)	74 (97%)
Squamous carcinoma	4 (5%)	0
Adenosquamous carcinoma	2 (2%)	2 (3%)
Other	0	0
***EGFR* sensitive mutation status by NGS**		
Exon 19 deletion	50 (53%)	36 (47%)
Exon 21 L858R	45 (47%)	40 (53%)

Data are median(range) or number of patients (%)
ECOG = Eastern Cooperative Oncology Group

Supplementary Table 2. Multivariate cox proportional hazard regression of predictive biomarkers and clinical status

	Coefficient	HR (95% CI)	Standard error of coefficient	z	p value	
RB1						
RB1 alterations	-0.404	0.67 (0.35-1.26)	0.3243	-1.247	0.21	
Treatments (Gefitinib vs VP)	-0.959	0.38 (0.24-0.603)	0.2317	-4.141	$3.46*10^{-5}$	***
Interaction between treatments and *RB1* alterations	1.376	3.96 (1.51-10.4)	0.4909	2.803	0.005	**
Age	-0.00047	1.00 (0.98-1.02)	0.0117	-0.040	0.97	
Sex	0.277	1.32 (0.75-2.32)	0.2885	0.962	0.34	
Smoking history	0.302	1.35 (0.72-2.54)	0.3222	0.938	0.35	
Clinical stage	0.088	1.09 (0.22-5.38)	0.8142	0.108	0.91	
N stage	0.496	1.64 (0.34-7.97)	0.8064	0.615	0.54	
NKX2-1						
NKX2-1 copy number gain	1.158	3.18 (1.69-6.02)	0.3246	3.568	$3.60*10^{-4}$	***
Treatment (Gefitinib vs VP)	-0.430	0.65 (0.42-1.02)	0.2280	-1.888	0.059	.
Interaction between treatment and *NKX2-1* copy number gain	-1.410	0.24 (0.091-0.66)	0.5057	-2.789	0.005	**
Age	-0.010	0.99 (0.97-1.01)	0.0115	-0.855	0.39	
Sex	0.272	1.31 (0.75-2.3)	0.2855	0.952	0.34	
Smoking history	0.234	1.26 (0.68-2.36)	0.3187	0.733	0.46	
Clinical stage	-0.038	0.96 (0.21-4.39)	0.7737	-0.049	0.96	
N stage	0.617	1.85 (0.41-8.31)	0.7659	0.805	0.42	
CDK4						

	Coefficient	HR (95% CI)	Standard error of coefficient	z	p value	
CDK4 copy number gain	0.852	2.34 (0.91-6.02)	0.4811	1.772	0.076	.
Treatment (Gefitinib vs VP)	-0.591	0.55 (0.37-0.83)	0.2081	-2.839	0.005	**
Interaction between treatment and *CDK4* copy number gain	-1.933	0.15 (0.026-0.81)	0.8792	-2.199	0.028	*
Age	-0.003	1.00 (0.98-1.02)	0.0115	-0.225	0.82	
Sex	0.300	1.35 (0.77-2.38)	0.2897	1.037	0.30	
Smoking history	0.300	1.35 (0.72-2.53)	0.3215	0.932	0.35	
Clinical stage	-0.021	0.98 (0.22-4.4)	0.7667	-0.027	0.98	
N stage	0.599	1.82 (0.41-8.07)	0.7596	0.789	0.43	
TP53						
TP53 exon4/5 missense mut	1.634	5.12 (2.34-11.2)	0.4002	4.082	$4.46*10^{-5}$	***
Treatment (Gefitinib vs VP)	-0.673	0.51 (0.33-0.80)	0.2262	-2.976	0.003	**
Interaction between treatment and *TP53* exon4/5 missense mutations	-1.023	0.36 (0.13-1.01)	0.5282	-1.937	0.053	.
Age	-0.003	1.00 (0.98-1.02)	0.0116	-0.219	0.83	
Sex	0.319	1.38 (0.78-2.43)	0.2910	1.096	0.27	
Smoking history	0.362	1.44 (0.76-2.71)	0.3234	1.120	0.26	
Clinical stage	0.075	1.08 (0.22-5.26)	0.8086	0.092	0.93	
N stage	0.525	1.69 (0.35-8.09)	0.7988	0.657	0.51	
MYC						
MYC copy number gain	-0.035	0.97 (0.34-2.73)	0.5295	-0.065	0.95	
Treatments (Gefitinib vs VP)	-0.575	0.56 (0.38-0.85)	0.2074	-2.773	0.006	**

	Coefficient	HR (95% CI)	Standard error of coefficient	z	p value	
Interaction between treatments and *MYC* copy number gain	-2.390	0.092 (0.0094-0.90)	1.1629	-2.055	0.04	*
Age	-0.006	0.99 (0.97-1.02)	0.0114	-0.525	0.60	
Sex	0.179	1.20 (0.67-2.13)	0.2943	0.607	0.54	
Smoking history	0.152	1.16 (0.61-2.22)	0.3304	0.460	0.65	
Clinical stage	-0.041	0.96 (0.22-4.20)	0.7531	-0.054	0.96	
N stage	0.666	1.95 (0.45-8.43)	0.7479	0.891	0.37	

VP, adjuvant chemotherapy regimen of vinorelbine plus cisplatin.

HR, hazard ratio;

CI, confidence interval;

P values are derived from two-sided wald test of the cox proportional hazards regression model.

Significant codes: ‘***’, p<0.001; ‘**’, p<0.01; ‘*’, p<0.05; ‘.’, p<0.1.

Supplementary Table 3. Interaction between treatments and *RB1* alteration

	Recurrence events/ No. of Patients	Coefficient	HR (95% CI)	Standard error of coefficient	*z*	*p* value
***RB1* mutations**	15/23	-0.34	0.71 (0.37-1.40)	0.343	-0.98	0.33
Treatments (Gefitinib vs VP)		-0.75	0.47 (0.31-0.72)	0.217	-3.47	$5.14*10^{-4}$ ***
Interaction between treatments and *RB1* mutations		1.21	3.34 (0.96-11.60)	0.634	1.90	0.06.
***RB1* copy number loss**	8/10	-0.39	0.68 (0.16-2.80)	0.724	-0.54	0.59
Treatments (Gefitinib vs VP)		-0.72	0.49 (0.33-0.73)	0.207	-3.45	$5.55*10^{-4}$ ***
Interaction between treatments and *RB1* copy number loss		1.35	3.86 (0.74-20.20)	0.844	1.60	0.11

VP, adjuvant chemotherapy regimen of vinorelbine plus cisplatin.

HR, hazard ratio;

CI, confidence interval;

P values are derived from two-sided wald test of the cox proportional hazards regression model.

Significant codes: '***', p<0.001; '**', p<0.01; '*', p<0.05; '.', p<0.1.

Supplementary Table 4. Multivariate cox proportional hazards regression of prognostic markers and clinical status

	Coefficient	HR (95% CI)	Standard error of coefficient	z	P value	
***TP53* exon 4-5 missense mut**						
TP53 exon 4-5 missense mut	0.989	2.69 (1.60-4.52)	0.2650	3.730	$1.91*10^{-4}$	***
Treatment (Gefitinib vs VP)	-0.850	0.43 (0.28-0.65)	0.2096	-4.040	$5.35*10^{-5}$	***
Age	-0.001	0.999 (0.98-1.02)	0.0117	-0.107	0.91	
Sex	0.278	1.32 (0.75-2.33)	0.2887	0.963	0.34	
Smoking history	0.333	1.39 (0.74-2.62)	0.3224	1.032	0.30	
Clinical stage	0.172	1.19 (0.23-6.07)	0.8321	0.207	0.84	
Lymph node status	0.463	1.59 (0.32-7.96)	0.8221	0.563	0.57	
***TP53* frameshift & truncating mut**						
TP53 frameshift & truncating mut	0.525	1.69 (1.08-2.65)	0.2294	2.290	0.022	*
Treatment (Gefitinib vs VP)	-0.676	0.51 (0.34-0.76)	0.2023	-3.341	$8.36*10^{-4}$	***
Age	-0.001	0.999 (0.98-1.02)	0.0115	-0.070	0.94	
Sex	0.280	1.32 (0.75-2.33)	0.2894	0.967	0.33	
Smoking history	0.187	1.21 (0.63-2.31)	0.3313	0.564	0.57	
Clinical stage	0.147	1.16 (0.27-5.01)	0.7475	0.197	0.84	
Lymph node status	0.405	1.50 (0.35-6.42)	0.7426	0.545	0.59	
***PIK3CA* mut**						
PIK3CA mut	-0.777	0.46 (0.24-0.88)	0.3312	-2.347	0.019	*
Treatment (Gefitinib vs VP)	-0.687	0.50 (0.34-0.75)	0.2026	-3.392	$6.93*10^{-4}$	***
Age	0.001	1.00 (0.98-1.02)	0.0114	0.085	0.93	

Sex	0.219	1.24 (0.70-2.21)	0.2937	0.745	0.46	
Smoking history	0.218	1.24 (0.65-2.36)	0.3280	0.663	0.51	
Clinical stage	0.581	1.79 (0.32-10.10)	0.8849	0.656	0.51	
Lymph node status	0.005	1.01 (0.18-5.61)	0.8771	0.006	1.00	
***MYC* gain**						
MYC gain	-1.047	0.35 (0.14-0.89)	0.473	-2.214	0.027	*
Treatment (Gefitinib vs VP)	-0.670	0.51 (0.34-0.76)	0.202	-3.308	$9.39*10^{-4}$	***
Age	-0.005	1.00 (0.97-1.02)	0.011	-0.399	0.690	
Sex	0.248	1.28 (0.73-2.26)	0.290	0.855	0.393	
Smoking history	0.279	1.32 (0.70-2.49)	0.323	0.864	0.388	
Clinical stage	-0.013	0.99 (0.22-4.35)	0.757	-0.017	0.986	
Lymph node status	0.609	1.84 (0.42-8.01)	0.751	0.812	0.417	
***NOTCH1* mut**						
NOTCH1 mut	0.907	2.48 (1.12-5.47)	0.404	2.244	0.025	*
Treatment (Gefitinib vs VP)	-0.714	0.49 (0.33-0.73)	0.203	-3.527	$4.20*10^{-4}$	***
Age	-0.003	1.00 (0.98-1.02)	0.011	-0.218	0.83	
Sex	0.391	1.48 (0.85-2.58)	0.284	1.375	0.17	
Smoking history	0.449	1.57 (0.84-2.92)	0.317	1.415	0.16	
Clinical stage	0.054	1.06 (0.23-4.91)	0.784	0.069	0.95	
Lymph node status	0.517	1.68 (0.37-7.7)	0.777	0.665	0.51	

VP, adjuvant chemotherapy regimen of vinorelbine plus cisplatin.

HR, hazard ratio;

CI, confidence interval;

P values are derived from two-sided wald test of the cox proportional hazards regression model.

Significant codes: ‘***’, p<0.001; ‘**’, p<0.01; ‘*’, p<0.05; ‘.’, p<0.1.
