# Supplementary material for: Genomic signatures define three subtypes of EGFR-mutant stage II–III non-small-cell lung cancer with distinct adjuvant therapy outcomes
Source: Nat Commun. 2021 Nov 8;12:6450. doi: 10.1038/s41467-021-26806-7 (PMC8575965; doi:10.1038/s41467-021-26806-7)
Supplement: Supplementary file 2 — Reporting Summary [file 41467_2021_26806_MOESM2_ESM.pdf]

nature portfolio

Corresponding author(s): Yi-Long Wu

Last updated by author(s): Jul 13, 2021

Reporting Summary

Nature Portfolio wishes to improve the reproducibility of the work that we publish. This form provides structure for consistency and transparency in reporting. For further information on Nature Portfolio policies, see our Editorial Policies and the Editorial Policy Checklist.

Statistics

For all statistical analyses, confirm that the following items are present in the figure legend, table legend, main text, or Methods section.

n/a	Confirmed	
☐	☒	The exact sample size (n) for each experimental group/condition, given as a discrete number and unit of measurement
☐	☒	A statement on whether measurements were taken from distinct samples or whether the same sample was measured repeatedly
☐	☒	The statistical test(s) used AND whether they are one- or two-sided<br>*Only common tests should be described solely by name; describe more complex techniques in the Methods section.*
☐	☒	A description of all covariates tested
☐	☒	A description of any assumptions or corrections, such as tests of normality and adjustment for multiple comparisons
☐	☒	A full description of the statistical parameters including central tendency (e.g. means) or other basic estimates (e.g. regression coefficient) AND variation (e.g. standard deviation) or associated estimates of uncertainty (e.g. confidence intervals)
☐	☒	For null hypothesis testing, the test statistic (e.g. F, t, r) with confidence intervals, effect sizes, degrees of freedom and P value noted<br>*Give P values as exact values whenever suitable.*
☒	☐	For Bayesian analysis, information on the choice of priors and Markov chain Monte Carlo settings
☒	☐	For hierarchical and complex designs, identification of the appropriate level for tests and full reporting of outcomes
☒	☐	Estimates of effect sizes (e.g. Cohen's d, Pearson's r), indicating how they were calculated

Our web collection on statistics for biologists contains articles on many of the points above.

Software and code

Policy information about availability of computer code

Data collection	No software was used for data collection.
Data analysis	Sequencing data was analyzed using Trimmomatic, Burrows-Wheller Aligner (v0.7.12), Genome Analysis Toolkit (GATK 3.4.0), and VarScan2. All statistical analysis were performed in R version 3.5.0. The custom code developed in the study is deposited in the Github repository at https://github.com/cancer-oncogenomics/minerva-adjuvant-nsclc (DOI: 10.5281/zenodo.5242512)

For manuscripts utilizing custom algorithms or software that are central to the research but not yet described in published literature, software must be made available to editors and reviewers. We strongly encourage code deposition in a community repository (e.g. GitHub). See the Nature Portfolio guidelines for submitting code & software for further information.

Data

Policy information about availability of data

All manuscripts must include a data availability statement. This statement should provide the following information, where applicable:

- Accession codes, unique identifiers, or web links for publicly available datasets
- A description of any restrictions on data availability
- For clinical datasets or third party data, please ensure that the statement adheres to our policy

The raw sequencing data generated in this study have been deposited in the National Omics Data Encyclopedia (NODE) database, hosted by the Bio-Med Big Data Center (BMDC) under accession code OEP002673. The raw sequencing data are available under restricted access due to data privacy laws. Access can be obtained by contacting corresponding authors of this study. The processed data matrices have been deposited in GitHub in the repository https://github.com/cancer-oncogenomics/minerva-adjuvant-nsclc (DOI: 10.5281/zenodo.5242512). The data generated from cross validation procedures in this study are provided in the Supplementary Information as Source Data file.

Field-specific reporting

Please select the one below that is the best fit for your research. If you are not sure, read the appropriate sections before making your selection.

☒ Life sciences ☐ Behavioural & social sciences ☐ Ecological, evolutionary & environmental sciences

For a reference copy of the document with all sections, see nature.com/documents/nr-reporting-summary-flat.pdf

Life sciences study design

All studies must disclose on these points even when the disclosure is negative.

Sample size	This study collected archived formalin-fixed paraffin-embedded (FFPE) tumor tissue specimens from the ADJUVANT/CTONG1104 trial. As a retrospective biomarker analysis, only patients with sufficient and qualified tumor tissue samples were included in this study. For NGS testing, we required a minimum of 5 FFPE sections or minimum DNA input of 250ng. Under this criteria, a total of 171 patients positive for EGFR by NGS were included. For independent validation, we obtained 37 FFPE pretreatment samples at the Guangdong Provincial People's Hospital which we have access to. Of these, 29 patients were had enough samples for NGS testing and were therefore included in the final validation cohort.
Data exclusions	Samples tested negative for EGFR sensitive mutations were excluded to ensure a uniform cohort to analyze EGFR co-mutations.
Replication	No replication was applied to individual patient sample. Ten formalin fixed paraffin-embedded (FFPE) sections of each patient's surgical sample was used for genomic DNA extraction.
Randomization	No randomization was applied in this exploratory biomarker analysis. Patients were randomized to receive either adjuvant gefitinib or adjuvant vinorelbine/cisplatin in the ADJUVANT trial. To demonstrate control of clinical covariates, multivariate analyses were performed with the identified genomic biomarkers to show no affect on biomarkers' statistical significance.
Blinding	Blinding was considered not appropriate for the design of this exploratory study.

Reporting for specific materials, systems and methods

We require information from authors about some types of materials, experimental systems and methods used in many studies. Here, indicate whether each material, system or method listed is relevant to your study. If you are not sure if a list item applies to your research, read the appropriate section before selecting a response.

Materials & experimental systems

n/a	Involved in the study	
☒	☐	Antibodies
☒	☐	Eukaryotic cell lines
☒	☐	Palaeontology and archaeology
☒	☐	Animals and other organisms
☐	☒	Human research participants
☐	☒	Clinical data
☒	☐	Dual use research of concern

Methods

n/a	Involved in the study	
☒	☐	ChIP-seq
☒	☐	Flow cytometry
☒	☐	MRI-based neuroimaging

Human research participants

Policy information about studies involving human research participants

Population characteristics	(NCT01405079) Patients aged 18–75 years with completely resected (R0), stage II–IIIA (N1–N2), EGFR-mutant (exon 19 deletion or exon 21 Leu858Arg) NSCLC.<br>(NCT01407822) Patients 18 years of older with previously untreated resectable stage IIIA-N2 NSCLC, and tumor tissue testing showed EGFR exon 19 or 21 mutation.
Recruitment	We did not particularly recruit patients for this biomarker analysis. All patients were recruited and enrolled during the original clinical trials and resected tumor specimens were obtained for this study.
Ethics oversight	Written informed consent was obtained from all patients. This study was approved by the research ethics board of Guangdong Provincial People's Hospital and all other participating hospitals (including Fudan University Affiliated Zhongshan Hospital, Shanghai, China; Zhejiang Cancer Hospital, Hangzhou, China; Hunan Cancer Hospital, Changsha, China; The Affiliated Hospital of Medical College Qingdao University, Qingdao, China; Liaoning Cancer Hospital, Shenyang, China; Fujian Medical University Union Hospital, Fuzhou, China; Jilin Provincial Tumor Hospital, Changchun, China; Jiangsu Cancer Hospital, Nanjing, China; The People's Hospital of Peking University, Beijing, China; Shanghai Pulmonary Hospital, Tongji University, Shanghai, China; Tangdu Hospital, Xi'an, China; Peking University First Hospital, Beijing, China; Fujian Cancer Hospital, Fuzhou, China; Beijing Chest Hospital, Beijing, China; The First Hospital of China Medical University, Shenyang, China; Beijing Cancer Hospital, Beijing, China; Harbin Medical University Cancer Hospital, Harbin, China; West China Hospital of Sichuan University, Chengdu, China; Sichuan Cancer Hospital, Chengdu, China; The Northern Jiangsu People's Hospital, Yangzhou, China; The First Affiliated Hospital of Suzhou University, Suzhou, China).

Note that full information on the approval of the study protocol must also be provided in the manuscript.

Clinical data

Policy information about clinical studies

All manuscripts should comply with the ICMJEguidelines for publication of clinical research and a completedCONSORT checklist must be included with all submissions.

Clinical trial registration	NCT01405079; NCT01407822
Study protocol	(NCT01405079) Patients were randomly assigned to receive either 250 mg oral gefitinib once a day for 24 months or 25 mg/m² intravenous vinorelbine on day 1 and day 8 plus 75 mg/m² intravenous cisplatin on day 1 every 3 weeks for four cycles (12 weeks in total). Treatment started 21–42 days after complete resection and continued until study completion, disease relapse, death, or unacceptable toxic effects. Full study protocol could be found in the appendix of Zhong et el. (Lancet Oncology, 2018) available at https://linkinghub.elsevier.com/retrieve/pii/S1470-2045(17)30729-5<br><br>(NCT01407822) Patients were randomly assigned to receive erlotinib 150 mg/d (neoadjuvant therapy, 42 days; adjuvant therapy, up to 12 months) or gemcitabine 1,250 mg/m2 plus cisplatin 75 mg/m2 (neoadjuvant therapy, two cycles; adjuvant therapy, up to two cycles). Assessments were performed at 6 weeks and every 3 months postsurgery. Full study protocol could be found in the supplements of Zhong et al. (Journal of Clinical Oncology, 2019) available at https://ascopubs.org/doi/suppl/10.1200/JCO.19.00075
Data collection	(NCT01405079) Patient were screened and enrolled at 27 centers in China between Sept 19, 2011, and April 24, 2014. Patients were assessed for disease-free survival by chest CT scan and abdominal ultrasound every 3 months, brain MRI every 6 months, bone scan every 12 months from baseline until disease relapse or death (whichever comes first) for up to 3 years. The survival after 3 years will be followed up semi-annually with telephone.<br><br>(NCT01407822) Patients were screened and enrolled at 17 centers in China between December 5, 2011, and December 13, 2017. Patients underwent chest, abdomen, and bone CT scans; brain magnetic resonance imaging (preferred), positron emission tomography, or CT to exclude brain metastases; and examinations of cardiac function. Tumor response was evaluated by CT scan after 6 weeks of neoadjuvant therapy (days 43 to 49). Postoperatively, patients received long-term follow-up, including chest CT scan and abdominal ultrasound every 3 months (and an abdominal CT scan in the event of suggestive findings in abdominal ultrasound), brain magnetic resonance imaging every 6 months, and a bone scan every 12 months for up to 2 years. Survival data were followed up for 3 additional years. Patients who discontinued treatment ahead of schedule were followed for survival.
Outcomes	(NCT01405079) The primary endpoint was investigator-assessed disease-free survival, which we defined as time from randomization to documented disease relapse or death, whichever occurred first. Secondary endpoints were overall survival (time from randomization to death from any cause); 3-year disease-free survival, 5-year disease-free survival, and 5-year overall survival; safety and tolerability; and HRQoL.<br><br>(NCT01407822) The primary endpoint was to compare the objective response rate (ORR) of erlotinib versus gemcitabine/cisplatin neoadjuvant therapy in surgically IIIA-N2 with epidermal growth factor receptor (EGFR) exon 19 or 21 sensitive mutant non-small cell lung cancer (NSCLC). Secondary endpoints were downsating rates of pathological lymph nodes, pathologic complete response, progression-free survival (PFS), 3 year and 5 year overall survival rate, quality of life (QoL) and safety biomarkers.
